# Commensal bacterial glycosylation at the interface of host–bacteria interactions

**DOI:** 10.1080/19490976.2025.2545421

**Published:** 2025-08-14

**Authors:** Chao Lei, Ting Wang, Jingzhi Wang, Yi Tan, Zhongbin Deng

**Affiliations:** a Department of Surgery, Division of Immunotherapy, University of Louisville, Louisville, KY, USA; b Brown Cancer Center, University of Louisville, Louisville, KY, USA; c Department of Microbiology and Immunology, University of Louisville, Louisville, KY, USA; d Pediatric Research Institute, Department of Pediatrics, University of Louisville, Louisville, KY, USA

**Keywords:** Glycoprotein, glycolipids, capsular polysaccharides, metabolic disease, colitis

## Abstract

Commensal bacteria produce a diverse array of glycosylated molecules, including glycoproteins, glycolipids, peptidoglycan, capsular polysaccharides, and exopolysaccharides, which play fundamental roles in host–microbe interactions. Recent advances have highlighted the intricate mechanisms by which bacterial glycosylation contributes to immune regulation, epithelial barrier integrity, and microbial community stability, with implications for a range of conditions, including infectious diseases, chronic inflammatory disorders such as inflammatory bowel disease (IBD) and Alzheimer’s disease, and metabolic diseases such as diet-induced obesity. This review provides a comprehensive synthesis of historical and recent insights into commensal bacterial glycosylation, emphasizing its role as a key mediator of host-bacteria interactions and its broader impact on gut homeostasis and systemic health.

## Introduction

Glycosylation is a widespread and critical post-translational modification in which monosaccharides, oligosaccharides, or polysaccharides are covalently attached to proteins, lipids, or other molecular substrates. This modification profoundly influences the structure, stability, and functional properties of the modified molecules, playing an essential role in various cellular processes such as metabolism, signal transduction, and immune modulation.^1,2^

**Figure 1.**
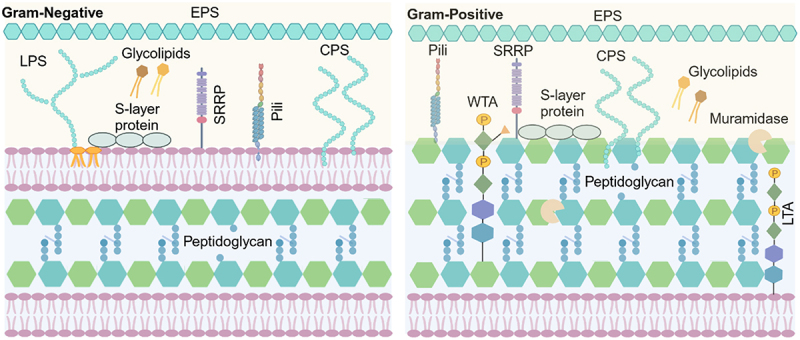
Overview of glycosylated surface structures in gram-negative and gram-positive bacteria involved in host interactions. Gram-negative bacteria (left) possess a double-membrane structure with an outer membrane containing lipopolysaccharides (LPS) and an inner cytoplasmic membrane, separated by a thin peptidoglycan (PG) layer in the periplasm. Glycosylated structures such as pili, serine-rich repeat proteins (SRRPs), S-layer proteins, and capsular polysaccharides (CPS) extend outward from the surface and mediate host adhesion and immune recognition. Gram-positive bacteria (right) lack an outer membrane but have a much thicker PG layer, into which teichoic acids are embedded. These include wall teichoic acids (WTA) covalently linked to PG and lipoteichoic acids (LTA) anchored in the cytoplasmic membrane. Glycoproteins such as SRRPs and S-layer proteins, along with glycosylated pili and CPS, are similarly exposed to the surface. Glycolipids and PG-associated enzymes like muramidases also contribute to structural remodeling and immune signaling. Exopolysaccharides (EPS) may form a biofilm-like matrix beyond the membrane and can be present on both gram types, forming an additional glycan-rich interface with the host.

While glycosylation was initially studied within the context of eukaryotic cells, it is now recognized as a ubiquitous and pivotal modification in all domains of life, including bacteria. Similar with eukaryotes, bacterial glycosylation occurs in various forms, including *N*- and O-linked glycosylation of glycoproteins, where glycans are covalently linked to serine/threonine, or asparagine residues. Unlike eukaryotic glycosylation, bacterial glycosylation is characterized by the incorporation of unique monosaccharides, such as pseudaminic acid, bacillosamine, N-acetylfucosamine, legionaminic acid, and 3-deoxy-D-manno-octulosonic acid.^3^ Additionally, bacterial glycosylation gives rise to distinctive glycan-containing structures, such as lipopolysaccharides, peptidoglycan, capsular polysaccharides, and exopolysaccharides, which are not found in eukaryotes.^4^ These unique features make bacterial glycosylation markedly more diverse and complex than its eukaryotic counterpart (Figure 1).

While the role of glycosylation in pathogenic bacteria has been extensively explored, particularly with regard to its contribution to pathogenicity, including bacterial colonization, immune evasion, and virulence factor expression,^5,6^ the study of glycosylation in commensal bacteria has lagged behind. However, the increasing recognition of the critical role of gut microbiota in maintaining host health has spurred a growing interest in understanding the molecular mechanisms underpinning host–microbe interactions, particularly in the context of commensal bacteria.

This review seeks to address this gap in knowledge by providing a comprehensive overview of both historical and contemporary advancements in the study of bacterial glycosylation, with an emphasis on its role in mediating interactions between commensal bacteria and the host. By highlighting the emerging functional implications of bacterial glycosylation in host health and immune homeostasis, this work aims to underscore its potential as a crucial factor in the dynamic interplay between host and microbiota.

## Glycoproteins

Bacteria produce numerous surface glycoproteins, including S-layer proteins, serine-rich repeat proteins, pili, flagella, and muramidases, all of which play critical roles in bacterial colonization, immune modulation, and disease pathogenesis (Table 1).

**Table 1.** Summary of glycosylated surface proteins discussed in this review.

Name	Species	Type	Sugar composition	Glycosylation type	Glycosylation function	Receptor/Target
SlpA	*Lactobacillus acidophilus*	SLP	Fuc, Man	O-glycosylation	CLR recognition and immune regulation	DC-SIGN/SIGN-R3
SLP-8321	*Lactobacillus kefiri*	SLP	Gal, Glc, Man	O-glycosylation	CLR recognition and immune regulation	Mincle
SLP-5818	*Lactobacillus kefiri*	SLP	Gal, Glc, Man	*N*- and O-glycosylation	CLR recognition and immune regulation	SIGN-R3
SLP-8348	*Lactobacillus kefiri*	SLP	Gal, Glc, Man	O-glycosylation	CLR recognition and immune regulation	Mincle
LspA	*Propionibacterium*	SLP	Man	O-glycosylation	CLR recognition and immune regulation	SIGN-R1
Srr1	*Streptococcus agalactiae*	SRRP	GlcNAc, Sia	O-glycosylation	Adhesion, translocation and stability	Keratin 4, Fibrinogen
SssP1	*Streptococcus suis*	SRRP	ND	O-glycosylation	ND	Vimentin
SRRP53608	*Lactobacillus reuteri* 53608	SRRP	Di-GlcNAc	O-glycosylation	Biofilm formation adhesion and colonization	ND
SRRP100–23	*Lactobacillus reuteri* 100–23	SRRP	Hex-Glc-GlcNAc	O-glycosylation	Biofilm formation adhesion and colonization	ND
SraP	*Staphylococcus aureus*	SRRP	ND	O-glycosylation	ND	sialylated receptors
SrpA/B/C	*Streptococcus salivarius*	SRRP	Hex, HexNAc, O-AcHexNAc	O-glycosylation	Adhesion and colonization	ND
PilE	*Neisseria meningitidis*	PILI	Gal, DATDH	O-glycosylation	Pilus biogenesis and aggregation	ND
PilA	*Pseudomonas aeruginosa*	PILI	O-antigen	O-glycosylation	Phage resistance	ND
SpaCBA	*Lactobacillus rhamnosus* GG	PILI	Fuc, Man	O-glycosylation	CLR recognition and immune regulation, adhesion	DC-SIGN
pilA	*Streptococcus agalactiae*	PILI	Sia	N-glycosylation	Stability and adhesion	ND
pilin	*Neisseria gonorrhoeae*	PILI	DATDH, Gal	O-glycosylation	CR3 recognition and activation	CR3 receptor
FliC1/FliC2	*Lactobacillus agilis*	Flagella	ND	O-glycosylation	Stability, motility and immune recognition	TLR5
flagellin	*Burkholderia cenocepacia*	Flagella	Qui4N(3‑OHBut)	O-glycosylation	Epithelial inflammatory responses	TLR5 recognition
Lc-p75	*Lacticaseibacillus casei*	Muramidase	ND	O-glycosylation	ND	ND
Acm2	*Lactobacillus plantarum*	Muramidase	HexNAc	O-glycosylation	Enzymatic activity	ND
Msp1/p75	*Lactobacillus rhamnosus* GG	Muramidase	Hex	O-glycosylation	Stability and translocation	ND

Abbreviations used in sugar composition: Glc, glucose; Gal, galactose; Man, mannose; Fuc, fucose; Sia, sialic acid; Hex, hexose; HexNAc, N-acetylhexosamine; GlcNAc, N-acetylglucosamine; o-AcHexAc, O-acetylated N-acetylhexosamine; DATDH, 2,3-diacetamido-2,3-dideoxyhexuronic acid; Qui4N(3‑OHBut), 4,6‑dideoxy‑4‑(3‑hydroxybutanoylamino)‑D‑ glucose; ND, not determined.

### S-Layer proteins

S-layer proteins form a crystalline, highly ordered two-dimensional lattice at the bacterial cell surface^7^. Typically composed of one or two major proteins, they function in cell wall attachment and self-assembly. S-layer glycoproteins exhibit considerable structural diversity and contribute to immune modulation and epithelial integrity, particularly in *Lactobacillus* species^8–17^.

S-layer proteins (SLPs) play a crucial role in bacterial interactions with the host immune system, with their glycosylation patterns determining recognition by specific C-type lectin receptors (CLRs). *Lactobacillus acidophilus* SlpA interacts with Dendritic Cell-Specific Intercellular adhesion molecule-3-Grabbing Non-integrin (DC-SIGN) through high-mannose and fucose residues, influencing dendritic cell activation and subsequent T cell differentiation^8,15^, supporting gut health and confer protection against colitis^18^. Similarly, studies on *Lactobacillus kefiri* have demonstrated that the immunostimulatory activity of SLP-8321 is dependent on recognition by Macrophage-inducible C-type lectin (Mincle), which binds specific microbial glycans and triggers innate immune signaling, while SLP-5818 is recognized by SignR3, the murine ortholog of human DC-SIGN^13,16,19^. These findings highlight the specificity of glycosylated SLPs in immune modulation. Notably, the loss of glycan integrity in *Lactobacillus kefiri* SLP-8348 significantly impairs its ability to elicit a cell-mediated immune response, further emphasizing the importance of glycosylation in immune recognition^13^. Moreover, the O-mannosylated large S-layer protein A (LspA) from *Propionibacterium* has been shown to interact with SIGNR1, thereby regulating dendritic cell function and intestinal immunity, enhancing resistance to *Listeria* infection and chemically induced colitis^20^.

### Serine-rich repeat proteins

Serine-rich repeat proteins (SRRPs) are a class of bacterial surface proteins characterized by a high serine content. SRRPs are often heavily glycosylated, a modification that influences their function and host interactions^21–31^.

The oral commensal *Streptococcus salivarius* utilizes SrpA, SrpB, and SrpC to facilitate colonization, with adhesion properties mediated by O-linked hexose, N-acetylglucosamine (GlcNAc), and O-acetyl-N-acetylhexosamine (O-AcHexNAc) glycans^22^. In *Lactobacillus reuteri*, SRRP glycosylation varies among strains, influencing biofilm formation and epithelial adhesion, with the SecA2/Y2 system-associated glycosyltransferases playing a crucial role in determining modification patterns^26^. Similarly, pathogens employ comparable strategies to enhance colonization and modulate virulence. For instance, in *Streptococcus agalactiae*, Srr-1, which is glycosylated with GlcNAc and sialic acid, binds Keratin 4 and Fibrinogen to promote epithelial adherence^23,30^. Glycosylation is critical for the adhesive and virulence functions of Srr-1, as blocking its GlcNAc residues with succinylated wheat germ agglutinin (sWGA) significantly reduces its binding capacity^30^. In *Streptococcus suis*, the fimbria-like glycoprotein SssP1 uses its immunoglobulin (Ig)-like domains to recognize sialic acid on host vimentin, promoting adhesion to host cells and contributing to virulence^21^. Likewise, in *Staphylococcus aureus*, SraP exhibits a lectin-like binding module that interacts with sialylated receptors, highlighting a conserved glycosylation-dependent mechanism for host interactions^25^.

### Pili

Bacterial pili are filamentous surface structures, typically consisting of multiple protein monomers and involved in adhesion, immune modulation, and phage resistance. Some pili are extensively glycosylated, which influences their interactions with host cells and immune responses^32–45^. For instance, the *Lactobacillus rhamnosus* GG SpaCBA pilus is glycosylated with mannose and fucose residues, recognized by dendritic cells via DC-SIGN, modulating cytokine responses^32,33,45^. Additionally, the mucus-binding pili of *L. rhamnosus* GG compete with *Enterococcus faecium* for adhesion sites, preventing pathogen colonization^36^. Multiple pathogenic bacteria employ similar strategies to invade host cells and evade immune defenses. The *Streptococcus agalactiae* NEM316 PilA protein is N-glycosylated and decorated with sialic acid, essential for PilA stability and host adhesion^41^. *Neisseria gonorrhoeae* pilin glycosylation modulates CR3 receptor activation, influencing immune signaling^37^. *Neisseria meningitidis* class II pilins (PilE) undergo extensive multisite glycosylation, which masks immune epitopes and facilitates immune evasion^35,40^. The type IV pili (PilA)of *Pseudomonas aeruginosa* function as phage receptors, with glycosylation providing a defense mechanism against pilus-specific phages^34^.

### Flagella

Bacterial flagella are helical, filamentous organelles that facilitate motility in many bacterial species. They are primarily composed of the protein flagellin and are anchored to the cell envelope by a complex basal body that spans the inner membrane, peptidoglycan layer, and, in Gram-negative bacteria, the outer membrane. In addition to their role in motility, flagella undergo glycosylation, which influences their structural stability, immune evasion, and interactions with the host. Most studies on flagellar glycosylation have focused on pathogenic bacteria^46–53^, with *Lactobacillus agilis* being the only commensal species in which flagellar glycosylation has been examined. *Lactobacillus agilis* produces glycosylated flagella composed of FliC1 and FliC2, with modifications affecting motility and immune recognition due to an incomplete TLR5-recognition site^54^. Similarly, the opportunistic pathogen *Burkholderia cenocepacia* flagellin is highly glycosylated, impairing epithelial inflammatory responses by reducing TLR5 recognition^50^.

### Muramidases

Muramidases are peptidoglycan hydrolases involved in bacterial cell wall remodeling. Glycosylation modulates their stability, enzymatic activity, and host interactions^55–65^. *Lactobacillus plantarum* Acm2 undergoes extensive O-glycosylation, with at least 15 N-acetylhexosamine (HexNAc) modifications, which regulate its binding affinity to peptidoglycan and hydrolytic efficiency^61,62^. Although Acm2 exhibits broad substrate specificity, its binding to peptidoglycan remains of relatively low affinity, with glucosamine serving as a minimal recognition motif^58^.

Another well-characterized muramidase, *Lactobacillus rhamnosus* GG Msp1/p75, is O-glycosylated at serine residues^60^. Msp1/P75 promotes gut homeostasis by activating Akt signaling, inhibiting apoptosis, and protecting epithelial barriers from TNF-induced damage. Msp1/p75 also enhances immune responses in Caco-2 cells via NF-κB and chemokine signaling, increasing CXCL1, CXCL8, and IL1B expression^64^. Additionally, it prevents oxidative stress-induced tight junction disruption through PKC and MAPK pathways^63,65^. Msp1/p75 modulates bile resistance by altering membrane accessibility to bile salts. Downregulation by lemon-derived exosomes enhances bile resistance and intestinal colonization, highlighting dietary regulation of bacterial adaptation^56,57^. Msp1/p75 also exhibits antifungal activity by inhibiting *Candida albicans* hyphal formation through chitinase-mediated chitin degradation, preventing adhesion and biofilm formation^55^. The glycosylation of Msp1/p75 seems to be important to its exportation, as only the cell wall associated and secretory Msp1/p75 are found to be glycosylated, but not the intracellular Msp1/p75^60^. Future research is needed to clarify the function of the saccharide moiety of Msp1/p75 in the interaction with a host. A homolog of Msp1/p75, Lc-p75 from *Lacticaseibacillus casei*, regulates immune responses by maintaining bacterial morphology. Its absence alters cell structure, impairing phagocytosis by moDCs, reducing cytokine secretion, and weakening Th1/Th17 activation^59^.

## Glycolipids

Bacteria produce a variety of glycolipids, including lipopolysaccharides, teichoic acids, lipoteichoic acids, α-galactosylceramides, and glucosyl-diacylglycerol, which play crucial roles in bacterial colonization, immune regulation, and virulence.

### Lipopolysaccharides (LPS)

**Figure 2.**
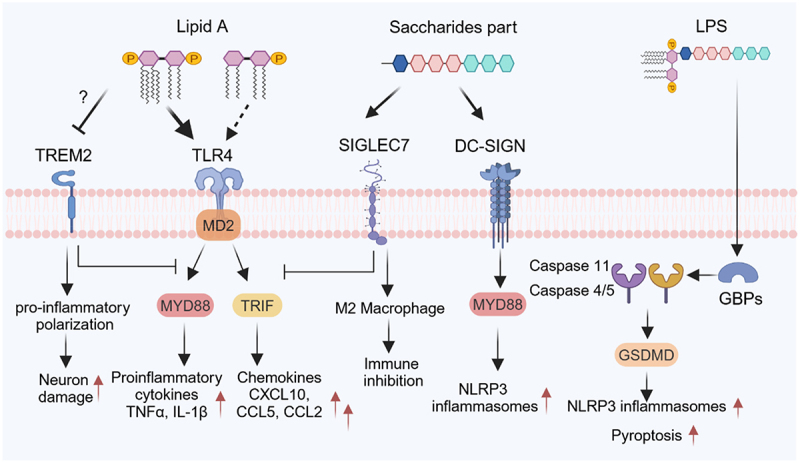
Immunoregulatory network of lipopolysaccharides (LPS). Lipid a structure dictates LPS immunogenicity, with hexa- or hepta-acylated, diphosphorylated lipid a serving as a toll-like receptor 4 (TLR4) agonist that, in conjunction with myeloid differentiation factor 2 (MD2), activates myeloid differentiation primary response 88 (MyD88)- and TIR-domain-containing adapter-inducing interferon-β (TRIF)-dependent signaling pathways, leading to pro-inflammatory responses. In contrast, tetra- or penta-acylated, monophosphorylated lipid a exhibits weak or negligible TLR4 activation. The core oligosaccharide and O-antigen moieties engage host receptors such as sialic acid-binding immunoglobulin-like lectin 7 (SIGLEC-7), promoting M2 macrophage differentiation and suppressing TLR4-mediated inflammation. O-antigen also interacts with dendritic cell-specific intercellular adhesion molecule-3-grabbing non-integrin (DC-SIGN), facilitating NLR family pyrin domain containing 3 (NLRP3) inflammasome activation. Cytosolic LPS is detected by guanylate-binding proteins (GBPs), leading to caspase-11 activation in mice or caspase-4/5 activation in humans. This leads to the cleavage of gasdermin D (GSDMD), promoting inflammasome assembly and pyroptosis. In addition, LPS suppresses the expression of triggering receptor expressed on myeloid cells 2 (TREM2) in microglia via an as-yet-unknown pathway, contributing to neuronal damage.

LPS, a key component of Gram-negative bacterial outer membranes, consists of three domains: the conserved lipid A moiety, a core oligosaccharide, and a highly variable O-antigen polysaccharide^66^. Lipid A serves as the primary immunostimulatory component, recognized by the TLR4/MD-2 complex, while the O-antigen contributes to structural diversity and immune evasion strategies (Figure 2).

#### Lipid a acylation and phosphate modifications in TLR4 activation

The lipid A moiety, comprising a β-1,6-linked glucosamine disaccharide bearing multiple acyl chains and phosphate groups, serves as the primary immunostimulatory component responsible for the endotoxic activity of LPS^67^. Hexa- or hepta-acylated LPS from *Proteobacteria* (e.g., *E. coli*, *Salmonella*) potently activates TLR4, triggering proinflammatory cytokine production (e.g., IL-1β, TNF-α) via the MyD88/TRIF pathway^67–69^. In contrast, commensal *Bacteroidetes* (e.g., Bacteroides) produce penta- or tetra-acylated LPS, which exhibits ~100-fold lower TLR4 agonism and can antagonize hexa-acylated LPS by competitively binding MD-2 and TLR4^70–79^. This structural divergence underlies the anti-inflammatory properties of commensal LPS, as evidenced by its ability to suppress *E. coli* LPS-induced inflammation^72,76^. Clinical studies further support this paradigm: healthy individuals exhibit a higher ratio of penta- to hexa-acylated LPS-producing bacteria compared to asthmatic patients^73^, suggesting a role for low-toxicity LPS in maintaining mucosal homeostasis^72,80^. In addition to the number of acyl chains, the length of lipid A acyl chains significantly influences TLR4 signaling strength. This has been observed in mutants of pathogenic bacteria lacking the late-stage lipid A acyltransferase HtrB/LpxL, which results in penta-acylated lipid A lacking C14:0 (myristate) and consequently leads to reduced inflammatory potential and virulence^81–84^. Compelling evidence from studies using engineered *Bordetella pertussis* strains with variable lipid A acyl chain lengths further demonstrates that shorter acyl chains diminish or abolish TLR4 activation, whereas longer acyl chains enhance it^85^.

The number and positioning of phosphate groups on lipid A further modulate LPS activity^70,71,86–88^. Monophosphoryl lipid A exhibits reduced endotoxicity compared to bisphosphorylated forms^70^. Intriguingly, even among penta-acylated LPS, phosphate localization dictates TLR4 engagement-*B. thetaiotaomicron* (1-phosphate) stimulates TLR4, whereas *Porphyromonas gingivalis* (4-phosphate) evades detection, highlighting the nuanced role of lipid A chemistry in immune evasion^71,86^.

Intriguingly, pathogenic bacteria actively modify the acylation and phosphorylation states of their lipid A in response to environmental cues, mimicking strategies used by commensal bacteria to maintain immune tolerance. This lipid A remodeling allows pathogens to evade immune detection or resist innate immune killing, highlighting the adaptive advantage of co-opting symbiosis-like mechanisms for pathogenesis^89,90^.

#### Cytosolic LPS and Non-canonical inflammasome activation

Beyond TLR4, cytosolic LPS is sensed by guanylate-binding proteins (GBPs), which recruit caspase-4/5 (humans) or caspase-11 (mice) to cleave gasdermin D (GSDMD), triggering pyroptosis and IL-1β release^91–96^. This GBP-caspase axis represents a critical defense mechanism against intracellular Gram-negative pathogens.

#### O-Antigen diversity and immune regulation

While less studied than lipid A, the O-antigen and core oligosaccharide exhibit structural plasticity that influences immune interactions^97,98^. For instance, *Fusobacterium nucleatum* LPS undergoes sialylation modifications that engage Siglec-7, inhibit TLR4 signaling and promote immune tolerance in tumor microenvironments^97^. Additionally, O-antigen length correlates with immunogenicity, as longer polysaccharides enhance vaccine efficacy^98^. DC-SIGN can also bind O-antigen^99^, though downstream signaling remains unclear.

#### LPS in barrier function and antimicrobial defense

Lipopolysaccharide (LPS) modulates gut barrier integrity and epithelial cell dynamics at physiological concentrations. By activating TLR4 signaling, LPS increases intestinal tight junction permeability without inducing cell death, while simultaneously suppressing intestinal stem cell proliferation and promoting apoptosis, mediated by TRIF and p53-upregulated modulator of apoptosis^100^. Notably, LPS derived from the cryptic specific core microbiota influences colonic epithelial cell fate, inhibiting proliferation and enhancing goblet cell differentiation via TLR4-dependent pathways^101^.

LPS also influences mucosal immunity^102–104^. *Alcaligenes*, a gut bacterium residing in dendritic cells of Peyer’s patches, potently induces IgA production with minimal inflammation^102,103^. Its LPS alone replicates this immunomodulatory activity despite weak TLR4 agonism, suggesting a unique role in shaping IgA responses.

LPS also serves as a critical target for host antimicrobial proteins/peptides (AMPs), shaping microbial-innate immune interactions. LPS induces the expression of small proline-rich protein 2A (SPRR2A), a bactericidal protein secreted by goblet and Paneth cells, through TLR4–MyD88 signaling^105^. On the other hand, LPS can also neutralize the membrane-disrupting activity of SPRR2A, allowing Gram-negative bacteria to evade SPRR2A-mediated killing^106^. These findings highlight the dual role of LPS in both promoting antimicrobial peptide (AMP) production and conferring resistance to AMPs, thereby giving Gram-negative bacteria a competitive advantage over Gram-positive species. Similarly, *Salmonella* modifies LPS via the PhoPQ system to induce host AMP LL-37 while simultaneously enhancing resistance to it, underscoring the importance of LPS phosphate groups in AMP interactions^107^. Structural studies reveal that 4-phosphate removal from *Bacteroides thetaiotaomicron* LPS reduces AMP resistance and impairs gut colonization during inflammation^108^, indicates that the 4-phosphate of LPS plays a critical role in conferring resistance to AMP.

#### LPS in neuroimmunological implications

Lipopolysaccharide (LPS) modulates microglial polarization and neuroinflammatory responses through its regulatory effects on triggering receptor expressed on myeloid cells 2 (TREM2)^109–112^. High-dose LPS suppresses TREM2 expression in microglia, disrupts the TLR4-TREM2 balance, promoting pro-inflammatory polarization, exacerbating neuroinflammation and neuronal damage, as seen in Alzheimer’s models^109^. Under pro-inflammatory conditions, including LPS exposure, TREM2 suppression impairs microglial survival and anti-inflammatory polarization, particularly in response to IL-4^110,112^.

### Teichoic acids

Teichoic acids (TAs), encompassing wall teichoic acid (WTA) and lipoteichoic acid (LTA), represent major structural components of Gram-positive bacterial cell walls, accounting for up to 60% of total cell wall mass. These anionic glycopolymers are characterized by repeating polyol-phosphate units (typically glycerol or ribitol phosphates) that confer a net negative charge^113^, enabling critical interactions with host surfaces and immune components. The structural diversity of TAs, including modifications such as D-alanylation and glycosylation, underlies their multifaceted roles in bacterial physiology and host–microbe interactions^114–123^.

#### TA in immune modulation

As major microbe-associated molecular patterns (MAMPs), TAs engage diverse immune recognition systems. LTAs from *Lactobacillus rhamnosus* GG activate NF-κB through TLR2/6 heterodimers^124^, while *L. plantarum* WTA induces IL-12 secretion via an actin remodeling-dependent, TLR2-independent pathway^125^. Notably, *S. aureus* β-GlcNAc-modified WTA specifically binds the langerin receptor on Langerhans cells, with β-GlcNAc-WTA showing superior binding affinity compared to α-GlcNAc-WTA^126^. Commensal-derived LTAs exhibit potent anti-inflammatory effects, with *L. plantarum* NCIMB8826 LTA inducing IL-10 production through TLR2-dependent signaling, an effect enhanced by D-alanine removal^118^.

TA structures critically influence adaptive immune responses. *Apilactobacillus kosoi* LTA, characterized by unique L-lysine modifications and absence of D-alanine substitutions, potently induces IgA production in Peyer’s patch cells^127^. Similarly, *L. plantarum* TA induces both effector and regulatory T cells in a D-alanylation-dependent manner^122^, while *S. epidermidis* TA activates CD8^+^ T cells via TLR2 signaling^128^. The zwitterionic WTA of *S. aureus* demonstrates MHC II-dependent T cell proliferative capacity, exceeding that of negatively charged WTAs or capsular polysaccharides^129^.

#### TA in gut homeostasis

The colonization capacity of Gram-positive bacteria is profoundly influenced by TA modifications. D-alanylation of TAs, mediated by the dlt operon, enhances resistance to cationic antimicrobial peptides (AMPs) through charge modulation, as demonstrated in *Lactobacillus plantarum* and *L. reuteri* 100–23^123,130^. Structural variations in the TA backbone, such as the glycerol-phosphate to ribitol-phosphate transition in *Staphylococcus epidermidis*, significantly impact colonization efficiency^117^. Pathogens including *S. aureus*^131,132^, *Listeria monocytogenes*^114^, and *Enterococcus faecalis*^133^ similarly exploit D-alanyl-TA modifications for host evasion and survival against both host-derived and microbial AMPs. Emerging evidence highlights the ability of TAs to directly influence epithelial function and gut barrier integrity. Lactobacillus-derived TAs have been shown to enhance digestive enzyme expression in Drosophila models^129^, while *Streptococcus thermophilus* TAs contribute to intestinal barrier maintenance in a D-alanylation-dependent manner^121^. These findings suggest that TA-mediated host modulation extends beyond classical immune recognition to include fundamental physiological processes.

### Other glycolipids

Glycolipids other than LPS and TAs serve as pivotal mediators in microbe-host interactions, modulating immune response and gut homeostasis.

#### Glycolipids in immune regulation

Cell wall glycolipids from *Corynebacterium diphtheriae* and *Corynebacterium ulcerans* directly engage Mincle. TLR2 is also required – both to upregulate Mincle expression and to achieve full macrophage activation through cooperative signaling^134^. Macrophages lacking Mincle or its adaptor protein, the Fc receptor gamma chain (FcRγ), exhibit markedly reduced production of granulocyte colony-stimulating factor (G-CSF) and nitric oxide (NO) in response to corynebacterial glycolipids. Mycolic acid esters, such as trehalose dimycolate, are critical immunogenic determinants, as strains lacking these glycolipids fail to trigger immune responses.

Invariant natural killer T (iNKT) cells are a unique subset of T lymphocytes that recognize lipid antigens presented by CD1d and play pivotal roles in bridging innate and adaptive immunity^135^. A series of studies have elucidated the structural and immunological basis by which iNKT cells respond to glycolipids derived from both commensal and pathogenic bacteria. In *B. fragilis*, sphingolipids (e.g., GSL-Bf717) suppress colonic iNKT cell populations, protecting against colitis by preventing excessive activation^136^. The α-galactosylceramides (BfaGCs) promote regulatory iNKT cell differentiation, inducing an IL-10-producing phenotype that dampens inflammation^137,138^. The sphinganine chain branching of BfaGCs, derived from branched-chain amino acids, dictates NKT cell activation specificity^139^. A functional metagenomic screen identified an α-glucosyl-diacylglycerol-producing glycosyltransferase in *B. fragilis* that antagonizes BfaGC-mediated NKT cell activation^140^, highlighting a lipid-driven regulatory axis controlling iNKT cell activity. Glycosphingolipids from *Sphingomonas spp*. activate invariant natural killer T (iNKT) cells via CD1d presentation, inducing cytokine production and innate immune activation independently of TLR4^141,142^. This pathway is essential for bacterial clearance, as iNKT-deficient mice exhibit impaired pathogen elimination. Pathogenic bacteria such as *Borrelia burgdorferi* and *Streptococcus* spp. produce diacylglycerol-based glycolipids that activate iNKT cells, with structural features such as lipid chain composition and CD1d-binding orientation critically determining antigenicity. These glycolipids engage iNKT cells either directly or through hepatic antigen-presenting cells like Kupffer cells, triggering rapid cytokine release and controlling systemic infection^143–146^.

#### Glycolipids and gut homeostasis

Gut bacteria produce a variety of glycolipids that contribute to gut homeostasis. *Lactobacillus johnsonii* produces structurally distinct glycolipids (GL1 and GL2) that interact with host immunity^147^. In inflammatory bowel disease (IBD) patients, disrupted tolerance to these commensal glycolipids is observed, marked by reduced anti-polysaccharide antibodies and elevated anti-glycolipid responses, suggesting their involvement in IBD pathogenesis^147^. GSL-Bf717 from *B. fragilis* also modulates gut motility by antagonizing α-GalCer-induced serotonin (5-HT) release in enterochromaffin cells via a CD1d-dependent mechanism^148^. *Enterococcus faecalis* diglucosyldiacylglycerol binds heparin on intestinal epithelial cells, promoting bacterial adhesion and translocation^149,150^. *Lactobacillus rhamnosus* glycolipid biosurfactants exhibit potent antibiofilm activity, disrupting pathogen membranes and colonization^151^.

## Peptidoglycan (PG)

**Figure 3.**
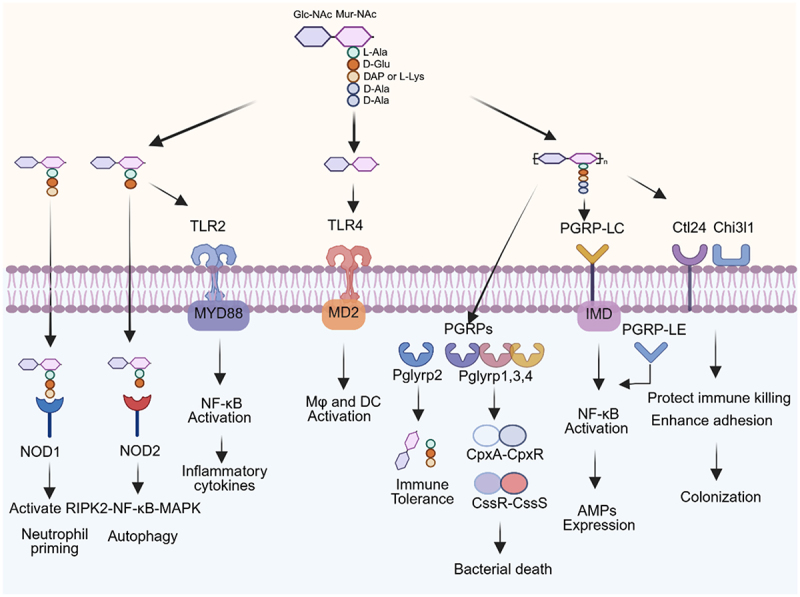
Peptidoglycan (PG) in host-microbe interactions.Peptidoglycan-derived muropeptides are recognized by intracellular nucleotide-binding oligomerization domain-containing protein 1 (NOD1/2) or extracellular toll-like receptor 2 (TLR2), leading to immune activation and the production of pro-inflammatory cytokines. The NOD1 signaling primes neutrophil responses, while the NOD2 signaling is normally associated with autophagy pathway. The disaccharide GlcNAc-MurNAc, derived from PG, engages the TLR4/MD2 pathway to activate macrophages and dendritic cells. Cytosolic PG is also recognized by peptidoglycan recognition proteins (PGRPs), which contribute to immune homeostasis by cleaving the PG to limit excessive inflammation (Pglyrp2 pathway) or directly inducing bacterial killing (Pglyrp1,3,4 pathway). In insects, the PG interacts with PGRP-LC to activate the expression of host antimicrobial proteins/peptides (AMPs). Additionally, PG can interact with host-derived proteins such as Ctl24 and Chi3l1, promoting bacterial colonization by enhancing adhesion and immune evasion. RIPK2: receptor-interacting serine/threonine-protein kinase 2, NF-Κb: nuclear factor kappa-light-chain-enhancer of activated B cells, MAPK: mitogen-activated protein kinase, Mφ: macrophage, DC: dendritic cell, IMD: immune deficiency pathway.

PG polymer consists of several linear glycan chains cross-linked to each other by short peptide chains. These glycan chains are composed of alternating units of two sugars, the NAG and the NAM, linked by β-1,4 glycosidic bonds. Short peptide chains attached to NAM residues cross-link adjacent glycan chains, creating a mesh-like structure^152^. PG is a complex polymer that represents an important structural component of the bacterial cell wall and plays a central role in shaping host-microbe interactions by acting as both an immune modulator and a structural determinant of bacterial physiology. It influences both innate and adaptive immunity through recognition by pattern recognition receptors (PRRs), modulation of inflammatory signaling pathways, and interactions with immune cells (Figure 3).

### PG recognition and immune modulation

PG fragments released by bacteria are detected primarily by nucleotide-binding oligomerization domain (NOD) proteins NOD1/2 and TLR2^153–161^. NOD1 specifically senses meso-diaminopimelic-type muropeptides from Gram-negative bacteria, while NOD2 acts as a more general sensor for various muropeptide structures^162^. Upon activation, NOD1/2 recruit RIPK2, triggering NF-κB and MAPK signaling cascades that drive the production of pro-inflammatory cytokines such as TNF-α, IL-6, and IL-8^158,159,163^. NOD1 recognition to PG systemically primes the innate immune system, enhancing immune killing to pathogenic bacteria^161^. NOD2 activates the autophagy pathway by recruiting ATG16L1 to the plasma membrane at bacterial entry sites^164^. Genetic variants that disrupt the NOD2-ATG16L1 interaction impair autophagy-mediated bacterial clearance and are strongly associated with increased susceptibility to Crohn’s disease^165^. Extracellular polymeric PG and PG derived fragments can also be recognized by TLR2 and activate TLR2 mediated immune response^69^. It is worth noting that the recognition of peptidoglycan (PG) by TLR2 remains controversial. Some studies have shown that highly purified PG or PG isolated from lipoprotein-lacking mutants (*Δlgt)* fails to activate TLR2^166–168^. However, other studies report that PG from *Δlgt* mutants retains TLR2-stimulating activity^169^. Muramidase treatment can abolish this activity^170^ and chemically synthesized muropeptides are still capable of binding to and activating TLR2^156^. NOD1/2 and TLR2 coordinate the intracellular and extracellular PG fragment sensing, playing a crucial role in eliminating invading bacteria and maintaining immune homeostasis. Notably, bacteria employ diverse strategies to recycle PG fragments, including the ABC transporter AmpG permease, the MppA-OppBCDF system, YtrBCDEF, and SLC transporters such as MurP and NagE^171,172^. These transporters facilitate the import of muropeptides from the periplasm or even the extracellular environment into the cytoplasm, thereby regulating the availability of immunostimulatory PG fragments to host immune cells. For example, the MppA – Opp system enables bacteria to scavenge extracellular peptides through the high-affinity binding of MppA (KD about 250 nM). By reducing the luminal concentration of muropeptides, this system helps suppress host immune activation, allowing bacteria to persist undetected during colonization^171^.

Peptidoglycan recognition proteins (PGRPs) also contribute to immune regulation by recognizing PG and functioning as amidases that hydrolyze PG or bactericidal proteins inducing oxidative and thiol stress to kill bacteria^173–176^. PGLYRP2, an N-acetylmuramoyl-l-alanine amidase, degrades PG before it can overstimulate immune responses, and its absence leads to excessive NOD2 activation and increased susceptibility to colitis^157^. PGLYRP1, PGLYRP3, and PGLYRP4 activate the CssR-CssS (gram-positive bacteria) and CpxA-CpxR (gram-negative bacteria) two-component system, which triggers a stress response that leads to bacterial death^175^. PGRP-LC, a transmembrane peptidoglycan recognition protein in insects, functions as a signal-transducing innate immune receptor. Together with PGRP-LE it activates NF-κB signaling and induces antimicrobial peptide gene expression in both systemic and local epithelial immune responses^177–180^.　A recent study demonstrated that PG-derived GlcNAc-MurNAc, a potential product of PGRPs, functions as a TLR4 agonist, activating macrophage and dendritic cell immune responses independently of NOD1/2 activation^181^.

Microbiota-derived PG influences systemic immunity, particularly through neutrophil priming via NOD1, which enhances resistance against bacterial infections^161^. Circulating PG fragments have been implicated in neuroimmune regulation, with gut-derived PG reaching distant organs and influencing brain inflammation, development, and behavior. Studies in *Drosophila* highlight PG signaling via PGRP-LC and Tak1 as crucial for neuronal innate immunity and synaptic plasticity^182^.

### PG in gut homeostasis

PG sensing is essential for intestinal barrier integrity, epithelial regeneration, and microbiota stability. NOD2 expression in intestinal stem cells connects PG signaling to epithelial health, with muramyl dipeptide supporting stem cell survival and tissue repair under stress^183^. PG from *Acetobacter persici* boosts stem cell proliferation and stress resistance through the PGRP-LC receptor^184^. Muropeptides from PG stimulate intestinal C-type lectin Ctl24, which recognizes peptidoglycan and coats bacteria, preventing over-elimination of bacteria by counteracting lysosome and anti-lipopolysaccharides factor B1 killing and supporting a stable microbial ecosystem^185^. Additionally, Chi3l1, secreted by intestinal cells, interacts with PG in the mucus layer and impacts microbiota^186^. A Chi3l1 deficiency leads to microbiota imbalance and worsens DSS-induced colitis.

## Capsular polysaccharides (CPS)

**Figure 4.**
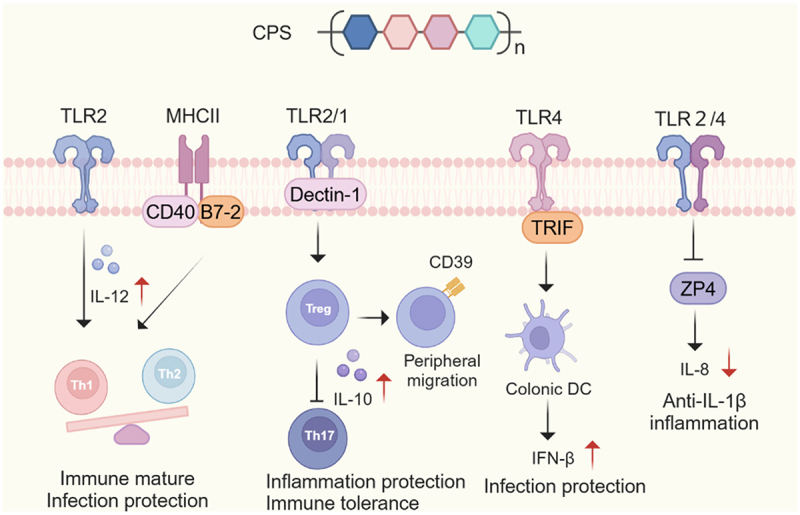
Capsular polysaccharides (CPS) in immune regulation.CPS play a critical role in modulating host immunity by promoting Th1 differentiation, thereby maintaining Th1/Th2 balance through TLR2- and major histocompatibility complex class II (MHCII)-dependent mechanisms. CPS also induce Tregs, suppressing Th17-driven inflammation and establishing immune tolerance. A distinct subset of CD39^+^ Tregs, induced by CPS, is essential for peripheral migration and systemic inflammation control. CPS-mediated activation of colonic dendritic cells further regulates virus immunity, while suppression of enterocyte-derived zona pellucida protein 4 (ZP4) via the TLR2/4 pathway contributes to inflammation control.

CPS are complex, high-molecular-weight carbohydrate structures that form a dense, organized layer tightly attached to the bacterial surface. During biosynthesis, these polysaccharides are typically covalently linked to lipid carriers and transported to the cell surface, where they assemble into a capsule^187,188^. Commensal bacterial CPS play key roles in colonization, immune modulation, gut barrier maintenance, and neuroimmune communication (Figure 4).

### CPS in microbial colonization and gut microbiota stability

CPS play a critical role in bacterial colonization by facilitating adhesion to host tissues and evading immune clearance. A well-characterized example is the capsular polysaccharide (CPS) produced by *Bacteroides fragilis*, a prominent human gut commensal. *B. fragilis* produces multiple CPS (PSA-PSH), with at least one capsular polysaccharide required for bacterial viability^189^. A complete polysaccharide repertoire is essential for *B. fragilis* colonization, as mutants expressing only a single, defined surface polysaccharide exhibit defective intestinal colonization compared to bacteria with a full complement of polysaccharides^189^. IgA-mediated mucosal binding enhances gut colonization by promoting CPS interactions with the host. PSB and PSC are critical for *B. fragilis* colonization due to their ability to bind IgA. Deleting global regulators such as *ccf* or removing the PSB/PSC structures significantly reduces IgA binding, leading to impaired *B. fragilis* colonization^190^. A recent study demonstrated that the removal of fucose from PSB, PSC, PSD, and PSH-achieved by deleting the fucosyltransferases in their respective biosynthesis clusters-reduces IgA binding to varying degrees^191^. This finding suggests that fucose-containing glycans play a key role in IgA coating and bacterial colonization. Consistently, complete loss of fucosylation, achieved by deleting both the de novo and salvage pathways of GDP-fucose biosynthesis, nearly abolishes IgA binding and markedly diminishes intestinal colonization^191^. Similarly, CPS contribute to ecological niche competition in *Bacteroides thetaiotaomicron*. The induction of high-affinity anti-CPS IgA in response to bacterial colonization strongly correlates with increased competition among strains expressing distinct CPS variants^192,193^.

Moreover, the zwitterionic PSA from *B. fragilis* inhibits the immune response through TLR2 signaling, promoting mucosal tolerance and reducing immune system-mediated clearance^194,195^. This mechanism facilitates the long-term persistence of *B. fragilis* in the gut.

However, CPS is not always beneficial for gut colonization. An inverse example is observed in *Bifidobacterium longum* 105-A, where CPS-D inhibits bacterial adhesion to enterocyte-like Caco-2 cells and reduces intestinal colonization by impairing fimbriae formation^196^.

### CPS in immune regulation

CPS from diverse bacterial sources exhibit widespread immunoregulatory activity. Among them, polysaccharide A (PSA) from *Bacteroides fragilis* is the most extensively studied, playing a crucial role in immune development, tolerance, and host-microbe symbiosis. PSA modulates the Th1/Th2 balance through TLR2 signaling, promoting a Th1 response in systemic immunity by inducing IL-12 production from antigen-presenting cells^197,198^. Similarly, CPS from *Bifidobacterium adolescentis* induces Th1-related immune responses while suppressing Th2 cytokine production in allergen-stimulated splenocytes^199^.

The balance between Th17 and Treg cells is crucial for mucosal immune homeostasis. PSA enhances Foxp3^+^ Treg differentiation and IL-10 secretion while suppressing Th17 responses, thus limiting IL-17-driven inflammation^194,195^. PSA recognition requires TLR2/TLR1 heterodimers, and Dectin-1 is necessary for PSA signaling and iNOS production^200^. Further studies have shown that B cell binding to PSA is essential for the induction of IL-10^+^ Tregs, which protect against viral encephalitis^201^. Similar effects on Tregs are observed with CPS from *Bifidobacterium bifidum* and *Bacteroides cellulosilyticus*, which use β-glucan/galactan (CSGG) polysaccharides and acetamido-amino-2,4,6-trideoxygalactose (AATGal)-containing ZPS to induce Tregs^202,203^.

PSA also mediates the tissue-specific expansion of a critical CD39^+^ CD4 Treg subset via the TLR2 signaling pathway^204,205^, contributing to protection against experimental autoimmune encephalomyelitis (EAE)^205^. CD39 is essential for Treg migration and function in the central nervous system (CNS), playing a role in suppressing neuroinflammation^206^.

CPS also modulate B cell activation and high-affinity IgA production^191,201^. Fucosylated CPS facilitates IgA coating, enabling interaction with GP2 on M cells, which aids bacterial entry into Peyer’s patches. This interaction activates germinal center B cells, plasma cells, and the production of high-affinity IgA^191^. Besides, the interaction of PSA with B cells induces IL-10-secreting regulatory B cells^201^, further promoting immune tolerance.

Dendritic cells (DCs) are also influenced by CPS, shaping immune responses at the mucosal interface. PSA activates plasmacytoid DCs (pDCs), leading to IL-10 secretion and the induction of a regulatory immune phenotype^207^. In neuroinflammatory conditions like EAE, PSA stimulation increases CD103 expression in DCs, promoting IL-10-producing Tregs and reducing CNS inflammation. PSA significantly enhances the ability of CD103^+^ DCs to convert naive CD4 T cells into IL-10-producing FoxP3^+^ Tregs in vitro, highlighting its role in regulating peripheral immune homeostasis^208^. A newly identified TLR4-TRIF pathway is involved in PSA-induced colonic DC activation, promoting IFN-β expression via TLR4-TRIF signaling and enhancing resistance to viral infections^209^.

### CPS in inflammation and disease modulation

CPS exhibits both pro- and anti-inflammatory properties depending on the bacterial strain and immune context. Zwitterionic polysaccharides (ZPS) from various bacteria elicit CD4 T cell responses through the MHC class II pathway^210,211^. ZPS’s unique ability to possess both positively and negatively charged motifs enables their processing and presentation by APCs, directly stimulating T cell activation and promoting Th1 differentiation, essential for intra-abdominal abscess development^212^. The costimulatory molecules B7–2 and CD40 play significant roles in ZPS-mediated T-cell activation^213^.

PSA exerts potent anti-inflammatory effects by inducing IL-10 production and preventing inflammation-associated diseases such as necrotizing enterocolitis (NEC), EAE, IBD, and colitis induced by *Helicobacter hepaticus* ^194,200,214–216^. PSA has also been shown to downregulate CCR5 expression in colitis-associated colorectal cancer models, mitigating inflammation-induced tumorigenesis^217^. Furthermore, PSA inhibits zona pellucida protein 4 (ZP4) in immature enterocytes, providing protection to IL-1β-induced inflammation, presenting potential therapeutic avenues for NEC^218^.

CPS demonstrate potential therapeutic effects to intestinal inflammation and colitis by enhancing gut barrier integrity^219,220^, restoring gut microbiota composition^219^, and modulating short-chain fatty acids (SCFAs) and phenylalanine metabolism^203,221^.

The CPS from *Ruminococcus gnavus* display a tolerogenic immune phenotype while masking pro-inflammatory antigens like glucose-rhamnose cell-wall polysaccharides, which are typically associated with inflammation^222^. This strategy is also employed by *Bifidobacterium bifidum*, which expresses anti-inflammatory β-glucan/galactan polysaccharides over pro-inflammatory phospho-glycero-β-galactofuranan polysaccharides^223^.

### CPS in intestinal neuron sensing

Emerging evidence suggests that CPS may directly influence gut sensory neurons, although the mechanisms remain unclear. PSA from *B. fragilis* has been shown to rapidly activate intestinal sensory neurons, indicating a potential role in inter-kingdom signaling between gut microbes and the nervous system^224^. These findings suggest that CPS may contribute to the regulation of the gut-brain axis, with implications for disorders such as irritable bowel syndrome (IBS) and neuroinflammatory diseases.

## Exopolysaccharides (EPS)

**Figure 5.**
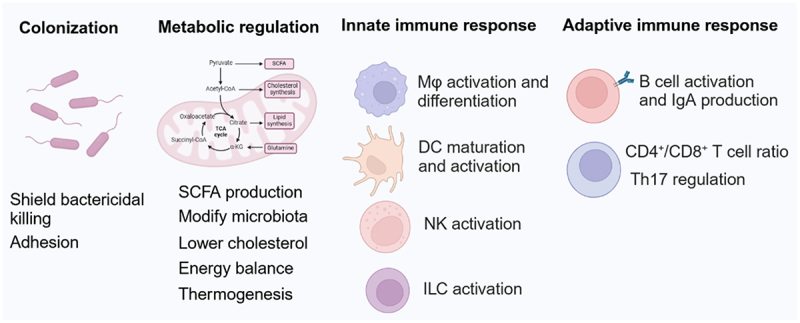
Exopolysaccharides (EPS) at the host-microbe interface.EPS influence bacterial colonization by shielding against bactericidal activity and modulating adhesion to host surfaces. Additionally, EPS contribute to metabolic homeostasis by reshaping gut microbiota composition, regulating short-chain fatty acids (SCFA) production, lowering serum cholesterol levels, and maintaining energy balance and thermogenesis. EPS also regulate both innate and adaptive immune responses through diverse mechanisms, thereby influencing host immunity and microbial community stability. Mφ: macrophage, DC: dendritic cell, NK: natural killer, ILC: innate lymphoid cells. Th17: T helper 17 cell.

EPS are high-molecular-weight carbohydrate polymers secreted by bacteria into the extracellular environment, typically forming a loose, protective matrix composed of either one type of monosaccharide (HoEPS) or multiple types (HeEPS) and often modified with non-carbohydrate substituents^225,226^. EPS produced by commensal and probiotic bacteria play crucial roles in modulating host immunity, maintaining gut homeostasis, and influencing host metabolism. These polysaccharides function as protective barriers, immunomodulators, and metabolic regulators, shaping microbe–host interactions through multiple mechanisms (Figure 5).

### EPS and bacterial persistence in the gut

EPS contributes to bacterial persistence by forming protective shields against host innate immune defenses^227–229^. For instance, the long galactose-rich EPS of *Lactobacillus rhamnosus* GG enhances bacterial survival by shielding against antimicrobial peptides and complement-mediated killing^227^. Similarly, *Bifidobacterium breve* UCC2003 exploits its surface EPS to evade host adaptive immunity and resist acidic and bile conditions, enhancing its persistence without affecting initial colonization^228^. Additionally, EPS from *Lactobacillus rhamnosus* KL 53A and *Lactobacillus casei* promote bacterial adhesion to the gastrointestinal epithelium, thereby facilitating colonization^229^.

### EPS and immune modulation

A key feature of EPS-mediated microbe–host interactions is immunomodulation. EPS from *Lactobacillus plantarum* induces dendritic cell maturation, promoting T cell proliferation and a pro-inflammatory immune response^230^. Rhamnose-rich hetero-exopolysaccharide from *Lactobacillus paracasei* DG activates THP-1 human monocytic cells, increasing gene expression of TNF-α, IL-6, IL-8, and CCL20 in macrophages^231^. Similarly, *L. rhamnosus* KL37 EPS stimulates cytokine production in peritoneal macrophages, though it is less effective than whole bacteria or LPS^232^. EPS from *Lactobacillus delbrueckii* ssp. *bulgaricus* OLL1073R–1 enhances NK cell activity and induces IFN-γ production through IL-12 and IL-18 signaling pathways, reducing the risk of cold and flu infections^233^.

Conversely, certain EPS exhibit anti-inflammatory effects. EPS from *Bacillus subtilis* promotes the differentiation of anti-inflammatory M2 macrophages, suppressing T cell activation through TGF-β and PD-L1 in a cell contact-dependent manner, providing protection against *Citrobacter rodentium*-induced colitis^234^. This EPS also induces IDO^+^ inhibitory dendritic cells via the TLR4-MyD88/TRIF pathway, preventing the activation of alloreactive T cells^235^. Likewise, EPS from *Bifidobacterium longum* 35624 dampens host inflammatory responses by repressing local Th17 cell activation^236^. The immunoregulatory properties of EPS extend beyond the gut, as demonstrated by *L. rhamnosus* KL37 EPS, which suppresses T cell-mediated arthritis in murine models^232^.

Moreover, EPS also influences B cell responses; for example, the absence of surface EPS on *B. breve* UCC2003 alters its antibody responses, as mice treated with wild-type UCC2003 exhibit lower antibody responses compared to those treated with EPS-negative *B. breve* ^237^. Additionally, exopolysaccharides produced by *Enterobacter cloacae* Z0206 enhance B lymphocyte proliferation and antioxidant enzyme activities^238^. *Leuconostoc mesenteroides* strain NTM048 EPS enhances fecal IgA production in a dose-dependent manner, increasing CD3^+^ T-cell and CD4^+^/CD8^+^ T　cell ratios^239^.

### EPS in host metabolism and metabolic disease prevention

Beyond immune modulation, EPS influences host metabolism and disease prevention. For instance, EPS from *Lactobacillus paracasei* and *Lactobacillus mucosae* lower serum cholesterol levels and modify gut microbiota composition in apoE-deficient mice, highlighting their potential cardiovascular benefits^240^. EPS from *Clostridium immunis*, modified with phosphocholine, regulates IL-22 levels via group 3 innate lymphoid cells, improving metabolic outcomes^241^. Similarly, *Streptococcus salivarius* EPS impacts SCFA production, energy balance, and thermogenesis, demonstrating a role in preventing diet-induced obesity^242^.

EPS also serves as a fermentable substrate for gut microbiota, leading to the production of SCFAs that contribute to host immune regulation and metabolic health. EPS from *Leuconostoc mesenteroides* alters gut microbial composition and promotes metabolic benefits^243^. Similarly, exopolysaccharides produced by intestinal *Bifidobacterium* strains act as fermentable substrates for human gut bacteria, significantly enhancing SCFA production^244^. Notably, *Lactobacillus paracasei* M7 EPS exhibits strong hypocholesterolemic and antioxidant activities, further emphasizing its metabolic benefits^245^.

### Additional functions of EPS in host health

Apart from their established functions in immune modulation and metabolic regulation, bacterial EPS have emerged as multifunctional mediators at the host–microbe interface. Recent studies reveal that EPS contribute to gut barrier integrity, cellular differentiation, gut-brain crosstalk, and tissue regeneration through diverse molecular mechanisms.

For example, EPS from *Bifidobacterium breve* plays a protective role in the intestinal epithelium by reducing apoptotic epithelial cell shedding via a MyD88-dependent signaling pathway, thereby supporting gut barrier integrity and mucosal homeostasis^246^. EPS from the psychobiotic strain *Lactobacillus fermentum* PS150 was identified as a key effector mediating its sleep-promoting effects. *In vivo* studies suggest that this EPS modulates the gut-brain axis, enhancing sleep duration and quality in mice, thus highlighting the influence of microbial EPS on host neurological function^247^. In the context of host development, EPS derived from *Enterococcus faecium* L15 has been shown to promote the osteogenic differentiation of human dental pulp stem cells through activation of the p38 MAPK pathway, indicating a potential role of bacterial EPS in modulating stem cell fate and tissue repair^248^. EPS produced by *Lactobacillus sp. Ca6* displays notable antioxidant and antibacterial properties and significantly accelerates dermal wound healing *in vivo*, suggesting a role in epithelial regeneration and barrier repair^249^.

Collectively, these findings underscore the multifaceted functions of bacterial EPS in shaping host physiology. EPS represent promising targets for future therapeutic strategies aimed at modulating gut microbiota – host interactions in both intestinal and systemic contexts.

## Summary and perspective

The interplay between commensal glycosylation and host biology underscores the significance of glycan-mediated interactions in host health. Bacteria utilize glycosylated structures such as glycoproteins, glycolipids, CPS and EPS to promote immune tolerance, support epithelial adherence, and spatially organize the microbial community. These glycan-mediated processes are central to maintaining symbiosis in the gut and ensuring host health. As research progresses, understanding the structural basis and functional consequences of bacterial glycosylation is becoming increasingly important.

Beyond their role in maintaining host-microbiota symbiosis, glycosylation strategies employed by commensal bacteria exhibit striking parallels to those used by pathogenic species to manipulate the host environment. For instance, capsular polysaccharides and glycoproteins that promote immune tolerance in symbionts may serve as immune evasion shields in pathogens such as Klebsiella pneumoniae and Mycobacterium tuberculosis, which utilize glycan masking and C-type lectin engagement (e.g., DC-SIGN) to suppress antigen presentation^250^. Likewise, *Helicobacter pylori* employs glycan mimicry (Lewis antigens) to evade TLR recognition and establish persistent colonization^251^. While glycosylated pili in commensals enhance epithelial barrier integrity and spatial organization, O-linked pilin glycan in Neisseria gonorrhoeae is required for binding to the I domain of complement receptor 3 on epithelial cells, promoting invasion^37^. Moreover, dynamic remodeling of glycan structures by commensals echoes pathogenic phase variation strategies. For example, the highly phase-variable capsule is essential for the virulence of *Campylobacter jejuni*^252^. These observations highlight a foundational duality: glycosylation serves as a conserved microbial toolkit for modulating host interactions-contextually tuned toward either mutualism or pathogenesis. Future comparative studies between commensals and pathogens may unveil targets to selectively bolster beneficial glycan functions while counteracting pathogenic mimicry.

While this review primarily focuses on the roles of glycosylated bacterial structures in host-microbe symbiosis, it is also important to highlight their broader functional significance in bacterial defense against environmental and biological threats. For instance, structural variations in O-antigen and CPS can prevent bacteriophage adsorption either by masking phage receptors or through phase-variable glycan remodeling, enabling evasion of phage predation^253–255^. Bacterial surface glycan structures, such as EPS also serve as protective barriers in biofilms, enhancing resistance to antibiotics, desiccation, and oxidative stress by limiting the penetration of harmful agents^256^. Additionally, glycosylation of cell wall components such as WTAs and LPS can modulate interactions with host-derived antimicrobial peptides and innate immune receptors^257–260^. These examples highlight how bacterial glycosylation not only contributes to host interaction but also represents a versatile and dynamic defense strategy that supports bacterial survival under selective pressures.

Another important and often underappreciated aspect of bacterial glycosylation is the extensive diversity that exists both at the genomic level (homologues) and within bacterial populations. For instance, *Neisseria meningitidis* strains expressing class II pili demonstrate significant polymorphisms within the pilin glycosylation (pgl) locus, including the replacement of canonical genes such as pglB with pglB2, and the presence or absence of unique open reading frames like ORF8. These variations result in structural heterogeneity in pilin glycosylation, which directly impacts immune recognition and serum resistance^261^. Similarly, *B. fragilis* possesses multiple distinct capsular polysaccharide (CPS) biosynthesis loci-more than eight in some strains-which can be differentially expressed. This genomic redundancy provides functional backup and allows for environmental tuning of surface properties to support competitive colonization and immune modulation^262^. In certain *Bacteroides* species, including *B. thetaiotaomicron* and *B. fragilis*, the phase-variable expression of CPS loci is controlled by invertible promoters. Such regulation allows for the stochastic switching between distinct glycan profiles within a clonal population, thereby enabling immune evasion and ecological flexibility in the gut^192,255,263,264^. These findings collectively demonstrate that glycan heterogeneity-arising from either genetic polymorphism or phase variation-serves as a conserved and strategic mechanism for bacteria to balance immune visibility and symbiotic persistence within the host environment.

Glycosylation is a well-established regulatory mechanism in eukaryotic systems, where it modulates protein folding, stability, trafficking, and receptor–ligand interactions^265^. In contrast, bacterial protein glycosylation remains less well understood and is often regarded as structurally diverse and species-specific rather than functionally conserved^4^. Emerging evidence indicates that bacterial glycosylation is not merely decorative: it can influence protein localization, adhesion properties, immune evasion, and enzymatic activity. Unlike the relatively conserved eukaryotic glycosylation pathways, bacterial systems often use noncanonical sugars and glycosyltransferases, and are frequently coupled to phase variation, further complicating their functional characterization^4^. Thus, while eukaryotic glycosylation is tightly integrated with cellular machinery and systemic regulation, bacterial protein glycosylation appears to function as a highly adaptable mechanism for niche-specific survival and host modulation, deserving deeper investigation.

Despite growing recognition of the importance of bacterial glycosylation, significant limitations persist in our understanding of glycan function. One major challenge lies in the structural complexity and heterogeneity of bacterial surface glycans^4^. These polysaccharides often consist of diverse monosaccharide compositions, branching patterns, and non-carbohydrate modifications, making comprehensive structural elucidation technically demanding. Furthermore, functional studies have disproportionately focused on the protein or lipid components of glycosylated molecules, while the contribution of the glycan moieties themselves remains underexplored. In the case of glycoproteins, most research emphasizes the protein backbone, with limited investigation into how specific glycan structures modulate protein stability, localization, or host interactions. These knowledge gaps highlight the urgent need for innovative tools and interdisciplinary approaches to define the specific biological roles of bacterial glycans in host–microbe interactions.

Fortunately, recent technological advances are transforming our ability to dissect and manipulate bacterial glycans. Bioorthogonal metabolic glycan labeling (MGL) now allows real-time visualization and enrichment of bacterial glycoconjugates in complex communities, while newly developed smart glycan probes enable condition-responsive detection of glycan dynamics in living systems^266,267^. Complementarily, bacterial-specific lectins and engineered glycan-binding proteins provide high-precision tools for studying glycan distribution, species-level variation, and interactions with host immune effectors^268,269^. The emergence of synthetic glycobiology has provided a modular framework for rebuilding and reprogramming glycosylation pathways. By combining well-defined glycosyltransferases, synthetic sugar donors, and engineered glycosylation motifs, researchers have begun to construct programmable glycosylation systems in both living and cell-free platforms^270^. These open systems offer unparalleled control over glycan structure and biosynthetic flux, supporting rapid prototyping and production of functional glycoconjugates-including glycans with tailored immune properties or therapeutic potential.

These technologies also underscore why bacterial glycosylation is such an attractive therapeutic target. First, the structural diversity of bacterial glycans-built from over 700 largely prokaryote-specific monosaccharides-offers a rich source of selective targets for antibiotics, vaccines, and glycan-based diagnostics^271^. Second, the functional flexibility of bacterial glycans makes them ideal substrates for therapeutic interface design, where glycan editing can enhance vaccine immunogenicity, functionalize probiotics, or redirect host immune responses^272^. Third, bacterial glycans are increasingly recognized as dynamic regulators, not static cell surface decorations; for example, selective lectin–glycan interactions (e.g., ZG16B, Galectin-7) regulate bacterial growth, localization, and immune recognition in a context-specific manner^273,274^. Furthermore, the inter-individual and interspecies variability of bacterial glycosylation patterns positions them as emerging biomarkers for microbiome-based diagnostics and personalized interventions. High-throughput tools such as Lectin-Seq^269,275^ and MGL-based glycan profiling^266^ allow species-resolved glycan mapping within microbial communities, paving the way for precision microbiome medicine based on glycosylation signatures.

In summary, bacterial glycosylation represents a powerful but still underexplored layer of host–microbe communication. As enabling technologies in chemical biology and synthetic glycobiology continue to mature, the field is rapidly shifting from passive characterization to active glycan engineering. This evolution opens new opportunities to not only decode bacterial glycan functions, but also to design glycan-targeted therapeutics that modulate microbial behavior, restore immune balance, and improve human health.
